# Supplementary material for: Empirical methods for controlling false positives and estimating confidence in ChIP-Seq peaks
Source: BMC Bioinformatics. 2008 Dec 5;9:523. doi: 10.1186/1471-2105-9-523 (PMC2628906; doi:10.1186/1471-2105-9-523)
Supplement: Additional File 1 — A variety of html documents from the USeq web site detailing the available applications, their best usage, output file type descriptions, command line menus, etc. [file 1471-2105-9-523-S1.zip › USeqUserGuides/JavaDoc.zip!/JavaDoc/trans/main/OverlapCounter.html]

OverlapCounter


|  |  |  |
| --- | --- | --- |
| **Overview** \| **Package** \| **Class** \| **Use** \| **Tree** \| **Deprecated** \| **Index** \| **Help** | |  |
| **PREV CLASS**   **NEXT CLASS** | **FRAMES**    **NO FRAMES**     **All Classes** |  |
| SUMMARY: NESTED \| FIELD \| CONSTR \| METHOD | DETAIL: FIELD \| CONSTR \| METHOD |  |


---


## trans.main Class OverlapCounter

```
java.lang.Object
  trans.main.OverlapCounter
```

---

``` public class OverlapCounter extends java.lang.Object ```

Provides an intersection analysis on multiple Interval[]s.

---

**Constructor Summary**

`OverlapCounter(java.lang.String[] args)`


| **Method Summary** | |
| --- | --- |
| `void` | `findClosestPeaks(Interval[] one, Interval[] two, java.io.File fileOne, java.io.File fileTwo)` |
| `static int` | `findPeak(Interval i)`             Returns base position of highest scoring peak, if no peak uses middle of sub window if no sub window uses middle of interval |
| `static void` | `main(java.lang.String[] args)` |
| `boolean` | `overlap(Interval one, Interval two)`             checks to see if intervals overlap by the minimum maxGap, can set maxGap negative to require an overlap. |
| `int` | `overlapPeaks(Interval one, Interval two)`             Returns distance between top peaks. |
| `static java.lang.String` | `percent(int overlaps, int total)` |
| `static void` | `printDocs()` |
| `static void` | `printTable(java.lang.Object[][] t, java.lang.String[] names)` |
| `void` | `processArgs(java.lang.String[] args)`             This method will process each argument and assign any new varibles |
| `static Interval[]` | `subtractIntervals(Interval[] bigger, Interval[] smaller)`             Returns the difference between two interval arrays, be sure to get bigger and smaller correct. |
| `java.lang.String` | `testOverlap(Interval[] one, Interval[] two, java.io.File fileOne, java.io.File fileTwo)`             Returns the average % intersection. |

**Methods inherited from class java.lang.Object**

`equals, getClass, hashCode, notify, notifyAll, toString, wait, wait, wait`

**Constructor Detail**

### OverlapCounter

```
public OverlapCounter(java.lang.String[] args)
```


**Method Detail**

### printTable

```
public static void printTable(java.lang.Object[][] t,
                              java.lang.String[] names)
```

---


### findClosestPeaks

```
public void findClosestPeaks(Interval[] one,
                             Interval[] two,
                             java.io.File fileOne,
                             java.io.File fileTwo)
```

---


### overlapPeaks

```
public int overlapPeaks(Interval one,
                        Interval two)
```

:   Returns distance between top peaks.
    if no peak uses middle of sub window
    if no sub window uses middle of interval
    returns -1 if different chromosomes, 0 if direct match, 1 if immediately adjacent, 2 if 1bp gap...

---


### findPeak

```
public static int findPeak(Interval i)
```

:   Returns base position of highest scoring peak,
    if no peak uses middle of sub window
    if no sub window uses middle of interval

---


### overlap

```
public boolean overlap(Interval one,
                       Interval two)
```

:   checks to see if intervals overlap by the minimum maxGap, can set maxGap negative to require an overlap.

---


### testOverlap

```
public java.lang.String testOverlap(Interval[] one,
                                    Interval[] two,
                                    java.io.File fileOne,
                                    java.io.File fileTwo)
```

:   Returns the average % intersection.

---


### percent

```
public static java.lang.String percent(int overlaps,
                                       int total)
```

---


### subtractIntervals

```
public static Interval[] subtractIntervals(Interval[] bigger,
                                           Interval[] smaller)
```

:   Returns the difference between two interval arrays, be sure to get bigger and smaller correct.

---


### processArgs

```
public void processArgs(java.lang.String[] args)
```

:   This method will process each argument and assign any new varibles

---


### printDocs

```
public static void printDocs()
```

---


### main

```
public static void main(java.lang.String[] args)
```


---


|  |  |  |
| --- | --- | --- |
| **Overview** \| **Package** \| **Class** \| **Use** \| **Tree** \| **Deprecated** \| **Index** \| **Help** | |  |
| **PREV CLASS**   **NEXT CLASS** | **FRAMES**    **NO FRAMES**     **All Classes** |  |
| SUMMARY: NESTED \| FIELD \| CONSTR \| METHOD | DETAIL: FIELD \| CONSTR \| METHOD |  |


---
